# Improving the Polyphenol Profile, Antioxidant and Antiglycation Activity of Onion Outer Scales with Chamomile Treatment

**DOI:** 10.17113/ftb.64.03.26.9473

**Published:** 2026-09-15

**Authors:** Valerija Vujčić Bok, Sanja Gagić, Lucija Rubinić, Ivana Šola, Petra Kašnjar Perković, Nika Celinić, Sandra Jurić, Željan Maleš

**Affiliations:** 1 University of Zagreb Faculty of Pharmacy and Biochemistry, Department of Pharmaceutical Botany, A. Kovačića 1, 10000 Zagreb, Croatia; 2 University of Zagreb Faculty of Science, Department of Biology, Horvatovac 102a, 10000 Zagreb, Croatia; 3 University of Zagreb Faculty of Pharmacy and Biochemistry, Department of Organic Chemistry, A. Kovačića 1, 10000 Zagreb, Croatia

**Keywords:** *Allium cepa* L., *Matricaria chamomilla* L., inhibition of BSA glycation, antioxidant activity, *in vitro* digestion, polyphenols

## Abstract

**Research background:**

The outer scales of common onion (*Allium cepa *L.) are often discarded as biological waste but they can be an interesting source of polyphenols with antioxidant and antidiabetic properties that can be used in the food and pharmaceutical industries.

**Experimental approach:**

The aim of this study is to use dried chamomile (*Matricaria chamomilla *L.) flowers containing 6 % flavonoid apigenin in the form of a water extract to improve the polyphenol profile, and the antioxidant and antiglycation activity of onion outer scales. “Red Carmen” onion bulbs with roots were exposed to deionized water (control) and chamomile water extract (test treatment) in a phytotron (23 °C/16 h day/8 h dark). Fourier transform infrared (FTIR) analysis was performed on dried, pulverised outer scales and chamomile. Pure standards of polyphenols (apigenin, quercetin and caffeic acid) were also used for FTIR spectral comparison.

**Results and conclusions:**

Total hydroxycinnamic acids (THA), total flavonols (TFL), antioxidant activity (2,2'-azino-bis(3-ethylbenzothiazoline-6-sulfonic acid (ABTS) and Fe(III) reducing antioxidant power (FRAP)), and inhibition of bovine serum albumin (BSA) by onion scale extract were measured in the initial, salivary, gastric and intestinal phases. Chamomile treatment increased THA, TFL, antioxidant (FRAP) and antiglycation activity (BSA method) in almost all *in vitro* digestion phases. Treating onions with chamomile water extract is an effective method for enriching biological waste, as confirmed by FTIR analysis. Enriched onion scales are a good source of bioaccessible polyphenols with high antioxidant (ABTS>85 % and FRAP>95 %) and moderate antiglycation activity (43–62 %).

**Novelty and scientific contribution:**

The enrichment of the phytochemical composition and biological potential of onions highlights the potential applications of this technique for health and nutritional purposes. Since the method does not require genetic modification but relies on natural absorption mechanisms and stress induction, it could become a valuable tool for enriching the nutritional and therapeutic composition of herbs. These results provide a foundation for future research, which should further elucidate the mechanisms of transmission, long-term effects, and potential industrial applications of this phenomenon.

## INTRODUCTION

In modern society, where nutrition increasingly goes beyond its basic role of satisfying hunger and is becoming a key component in preventing certain diseases and preserving health, plants are no longer seen only as a source of nutrients but also as a rich reservoir of natural healing compounds. The growing consumer interest in functional foods and nutritionally rich, fresh, minimally processed products is driving research aimed at increasing their content of bioactive compounds such as flavonoids, glucosinolates, phenolic acids and vitamin C (*1*, *2*).

The composition and concentrations of bioactive substances in plants are known to change depending on environmental factors, but they can also be increased in a targeted manner using agrotechnical procedures and biotechnological methods, or by inducing stress conditions such as UV radiation and temperature changes (*3*, *4*). However, with these known approaches, in recent years increasing attention has been paid to a strategy that brings a new dimension to this field: the transfer of phytochemical compounds from one plant to another, the so-called interspecific transfer of plant metabolites. This phenomenon, also known as horizontal natural products transfer (HNPT), involves the movement of specialised (secondary) metabolites from donor plants to recipient plants, most often through the shared soil and root system (rhizosphere) (*5*), and it is assumed that compounds from the soil enter by passive diffusion through the plasmalemma of root cells (*6*). To confirm the possibility of such metabolite transfer, Selmar *et al*. (*7*) conducted a co-cultivation experiment in which they grew the plant species *Senecio jacobaea* L., which contains high concentrations of pyrrolizidine alkaloids (PA), together with parsley (*Petroselinum crispum* L.) in common pots. After two months of co-cultivation and analysis, significant concentrations of PA were found in parsley plants, averaging more than 200 μg/kg of dry matter. The parsley plant was therefore able to absorb pyrrolizidine alkaloids from the soil, even though it does not have the biosynthetic ability for these compounds itself.

A particularly important group of plant metabolites are flavonoids, phenolic acids and related polyphenolic compounds, which have numerous beneficial effects on human health (*8*, *9*). They are valued for their antioxidant, anti-inflammatory, antiproliferative and antidiabetic properties; however, their instability in the gastrointestinal tract and low bioavailability limit their full potential. Consequently, ways to increase their concentration in plants are being investigated, and in this context the model of interspecies transfer of metabolites appears to be a potentially powerful tool for improving the phytochemical profile. In addition to the previously mentioned transfers of metabolites through soil, more recent research has introduced another strategy: interspecific transfer *via* plant extracts. This was precisely the focus of the work of Šola *et al*. (*10*), which showed that the treatment of Chinese cabbage seedlings with extracts of plants such as chamomile, St. John's wort, rose and black bryony can significantly modify their phytochemical profile and biological activities, including antidiabetic and antioxidant potential. Although the authors cautiously consider whether this represents real transfer of compounds or an induction of biosynthesis in the recipient plant, the effect on the concentration of phenolic compounds and on antioxidant and antidiabetic activity was measurable and significant.

The greatest wealth of chamomile, as a guardian of traditional medicine, lies in its flower heads. These contain flavonoids such as apigenin, quercetin and luteolin, phenolic acids, including ferulic and caffeic acids, coumarins, and powerful essential oils with α-bisabolol and chamazulene as key components (*11*). Phenols exhibit antioxidant and sedative properties. It has traditionally been known as a "cure-all" and is officially recognised in 26 pharmacopoeias worldwide (*11*). It is used in the form of teas, extracts, tinctures, creams and baths. In recent years, its antioxidant and antidiabetic properties have attracted particular scientific attention. Apigenin, one of its main flavonoids, together with hydroxycinnamic acids, contributes to the regulation of blood glucose, so these compounds show exceptional potential in alleviating the symptoms of type 2 diabetes, improving lipid profiles, and preventing metabolic syndrome (*12*).

Among the most common groups of compounds in common onion bulbs and outer onion scales, there is a trio: organosulfur compounds, flavonoids and fructans. Sulfur compounds, responsible for the tears caused by onion cutting, mostly belong to the group of S-alk(en)yl-l-cysteine sulfoxides, among which isoalliin predominates. In addition to determining the taste and smell of onions, these compounds have antimicrobial, hypolipidaemic, antidiabetic and anti-inflammatory properties (*13*). Flavonoids, primarily quercetin, whose concentration in certain cultivars is as much as five to ten times higher than in broccoli, apples or blueberries (*14*), have antioxidant, anticancer and cardioprotective effects. In addition to quercetin, kaempferol and, in coloured varieties, anthocyanins are also present. Among the phenolic acids, ferulic, caffeic and gallic acids stand out (*15*). Fructans, especially inulin, which has a prebiotic effect, and numerous organic acids that contribute to the spiciness and stability of onions, should also not be neglected. Outer scales of common onion are often discarded as biological waste but can be an interesting source of polyphenols with antioxidant and antidiabetic properties that can be used in the food and pharmaceutical industries. According to Ellatar *et al*. (*16*), the diverse phytochemical composition of the outer scales, leaves and roots of the common onion has been demonstrated, and the importance of using onion waste parts as a source of valuable biologically active components has been emphasised. A total of 103 compounds were reported using UPLC-ESI-MS/MS, with flavonoids being the most abundant. Compounds from the groups of phenolic acids, organic acids, saponins, amino acids, organosulfur compounds and fatty acids were also detected (*16*).

In this study, the extract of chamomile (*Matricaria chamomilla* L.) served as a transfer medium for valuable phytoactive substances to the common onion (*Allium cepa* L.). After treatment with chamomile extract, changes in the content of flavonols and hydroxycinnamic acids in the onion outer scales were analysed. The biological activity of onions as an acceptor plant was also investigated, with particular emphasis on its altered antidiabetic and antioxidant functions. All analyses were performed using an *in vitro* model of human digestion that simulates the physiological conditions of the gastrointestinal tract (different pH in different parts of the digestive system; the presence of stomach acid, bile and digestive enzymes; different durations of the oral, gastric and intestinal digestion phases; simulation of intestinal peristalsis, *etc*.). Antidiabetic activity was measured by inhibition of fructose binding to the protein bovine serum albumin (BSA), which mimics physiological mechanisms associated with the development of diabetic complications, and antioxidant activity by the ABTS and FRAP methods. FTIR spectra of the powdered onion outer scales subjected to chamomile treatment or control treatment were recorded and compared with the FTIR spectra of the polyphenol standards apigenin, quercetin and caffeic acid and with the FTIR spectra of the powdered dried chamomile flowers.

## MATERIALS AND METHODS

### Chemicals and materials

All chemicals and reagents used were of analytical or HPLC grade. Enzymes required for the *in vitro* digestion protocol (α-amylase, porcine pepsin, pancreatic lipase and pancreatin), as well as bile extract and α-amylase used in the antidiabetic assay, were obtained from Merck KGaA (Darmstadt, Germany). Commercial polyphenol standards were purchased from Merck KGaA and Extrasynthese (Genay, France). Chemicals and solvents were provided by Merck KGaA or Kemika (Zagreb, Croatia), and deionized water was used throughout all experiments.

Absorbance and fluorescence measurements related to hydroxycinnamic acids and flavonols, and to antioxidant and antiglycation activity were recorded using a Fluostar Optima microplate reader (BMG Labtech GmbH, Ortenberg, Germany).

### Plant material

Dried chamomile (*Matricaria chamomilla* L.) flowers with a declared flavonoid content of 6 % apigenin were purchased from Galenic and Analytical Laboratories of the City Pharmacies Zagreb (Zagreb, Croatia). A mass of 3 g was infused with 250 mL of hot water and stirred for 60 min, after which the extract was filtered, cooled to room temperature, and then used for subsequent experiments. Bulbs of red onion (*Allium cepa* L. var. Red Carmen; Naktuinbouw, Roelofarendsveen, the Netherlands) were obtained from a local store in December 2024. Bulbs of uniform size (1.5–2.0 cm) and good health were selected, while dried adventitious roots were removed. For root induction, bulbs were placed in glass tubes with deionized water and incubated for 48 h in a Fito-Clima 600 PLH climate chamber (Aralab, Rio de Mouro, Portugal) at 23 °C and 65 % humidity, under a 16 h light/8 h dark regime. Only specimens with normally developed roots were used in experiments; bulbs with visible root malformations were excluded. For treatment, selected bulbs were incubated for 24 h either in deionized water (control) or in the aqueous chamomile extract (test). After incubation, outer scales were rinsed with water, dried, pulverised, and used for ethanolic extraction. Powdered onion scales were extracted with 70 % ethanol to yield a final concentration of 50 mg/mL, which was subsequently applied in the *in vitro* digestion assays.

### Extract preparation

Ethanolic extracts were prepared by suspending powdered onion outer scales in *φ*(ethanol)=70 % to a concentration of 50 mg/mL. Extractions were carried out at room temperature using a rotary extractor (Thermo Fisher Scientific, Shanghai, PR China) for 60 min. After centrifugation (9727×*g*, 5 min) in a laboratory centrifuge (MIKRO 220R centrifuge; Hettich, Tuttlingen, Germany), the supernatants were collected and stored at –20 °C until analysis.

### Model of human in vitro digestion

The *in vitro* digestion of the extract from onion outer scales was conducted following a modified procedure based on Vujčić Bok *et al*. (*17*). An aliquot of 0.15 mL extract was combined with 0.15 mL of 20 mmol/L phosphate buffer (pH=7.0). The oral (salivary) phase was initiated by adding 5 µL of α-amylase (0.48 mg/mL in the same buffer) and incubating the mixture for 5 min at 37 °C in a thermal shaker (HP 15A and TH15; Edmund Bühler, Tübingen, Germany) at 150 rpm.

To simulate gastric conditions, 0.2 mL of porcine pepsin solution (3 mg/mL in 0.1 mol/L HCl) was added. The pH was then adjusted to 2.0 with 1 mol/L HCl, and samples were incubated for 1 h at 37 °C with constant shaking.

For the intestinal phase, the pH was first raised to 5.3 by the addition of 5 µL of 1 mol/L NaHCO_3_. Pancreatic juice (0.45 mL), containing 2.4 mg/mL bile acids, 0.2 mg/mL porcine pancreatic lipase and 0.4 mg/mL pancreatin in 20 mmol/L phosphate buffer (pH=7.0), was then added. The total sample volume was brought to 1 mL with phosphate buffer, and the final pH was adjusted to 7.0 using 1 mol/L NaOH. Incubation continued for 2 h at 37 °C with shaking at 150 rpm.

After each digestion step, including the initial (pre-digestion) phase, the volume of samples was standardised to 1 mL with 20 mmol/L phosphate buffer (pH=7.0). All samples were centrifuged at 21 885×*g* for 5 min at 4 °C in laboratory centrifuge (MIKRO 220R centrifuge; Hettich), and supernatants were stored at –20 °C until further spectrophotometric fluorescence analysis.

### Phytochemical analysis

The total contents of hydroxycinnamic acids (THA) and flavonols (TFL) were quantified according to Howard *et al*. (*18*), using caffeic acid and quercetin as standards, respectively. Briefly, 50 µL of extract (7.5 mg/mL) were combined with 50 µL HCl (1 mg/mL in ethanol) and 0.91 mL HCl (2 mg/mL). Absorbance was read at 320 for THA and 360 nm for TFL. Results were expressed as milligrams of caffeic acid equivalents (CAE) per gram of dry mass (mg/g) or milligrams of quercetin equivalents (QE) per gram of extract (mg/g). Bioaccessibility (%) was determined as the ratio of compound concentration in each digestion phase to the initial undigested extract.

### Antioxidant and antiglycation activity

The antioxidant properties of the extracts were evaluated using 2,2’-azinobis(3-ethylbenzothiazoline-6-sulfonic acid) (ABTS) and Fe(III) reducing antioxidant power assays (FRAP). For the ABTS test (*19*), 2 µL of extract were mixed with 200 µL of ABTS solution and incubated at room temperature for 6 min. The decrease in absorbance was measured at 740 nm, and the percentage of ABTS radical inhibition was calculated.

The FRAP assay (*20*) involved combining 10 µL of extract with 190 µL of freshly prepared FRAP reagent. After 4 min of incubation at room temperature, absorbance at 595 nm was measured, and the reduction of Fe(III)-2,4,6-tris(2-pyridyl)-s-triazine was expressed as a percentage. Trolox served as the positive control for both antioxidant assays.

To determine antiglycation activity, BSA glycation was inhibited according to Rusak *et al*. (*21*). Samples containing 100 µL BSA solution (10 mg/mL), 100 µL fructose solution (0.5 mol/L), and 40 µL extract were incubated in a shaker incubator (HP 15A and TH15; Edmund Bühler) at 37 °C for 24 h. After incubation, fluorescence was measured at an excitation wavelength of 405 nm and emission wavelength of 460 nm. Catechin solution was used as a reference, and inhibitory activity was calculated accordingly.

### FTIR analysis

Powdered chamomile and onion outer scales (chamomile treatment or control) or the polyphenol were placed on the sample holder in a Fourier transform infrared (FTIR) spectrophotometer (Spectrum Two; PerkinElmer, Inc., Waltham, MA, USA) operated using Spectrum Touch software (PerkinElmer, Inc.). The absorption spectrum of each sample was measured in the range 4000 to 500 cm^-1^. A background scan with an empty sample plate was done before the analysis of each sample. Each treatment was measured in triplicate. The IR spectra were analysed by observing vibrations of sample atoms when they were exposed to the IR region of the electromagnetic spectrum.

### Statistical analysis

Data were processed with Statistica v. 13.3 software (*22*). One-way analysis of variance together with Duncan’s *post hoc* test was used to assess statistically significant differences (p≤0.05). Principal component analysis (PCA) was applied to visualise grouping patterns among samples. Pearson’s correlation coefficients were calculated to evaluate the relationships between total polyphenols and the measured biological activities, including antioxidant and antiglycation potential.

## RESULTS AND DISCUSSION

### Polyphenol profile, and antioxidant and antiglycation activities

Fig. 1 shows the total hydroxycinnamic acid (THA) and total flavonol (TFL) content in the control sample (deionized water) and the treated (chamomile) onion outer scales before (initial phase) and after (salivary, gastric and intestinal phases) *in vitro* digestion.

**Fig. 1:**
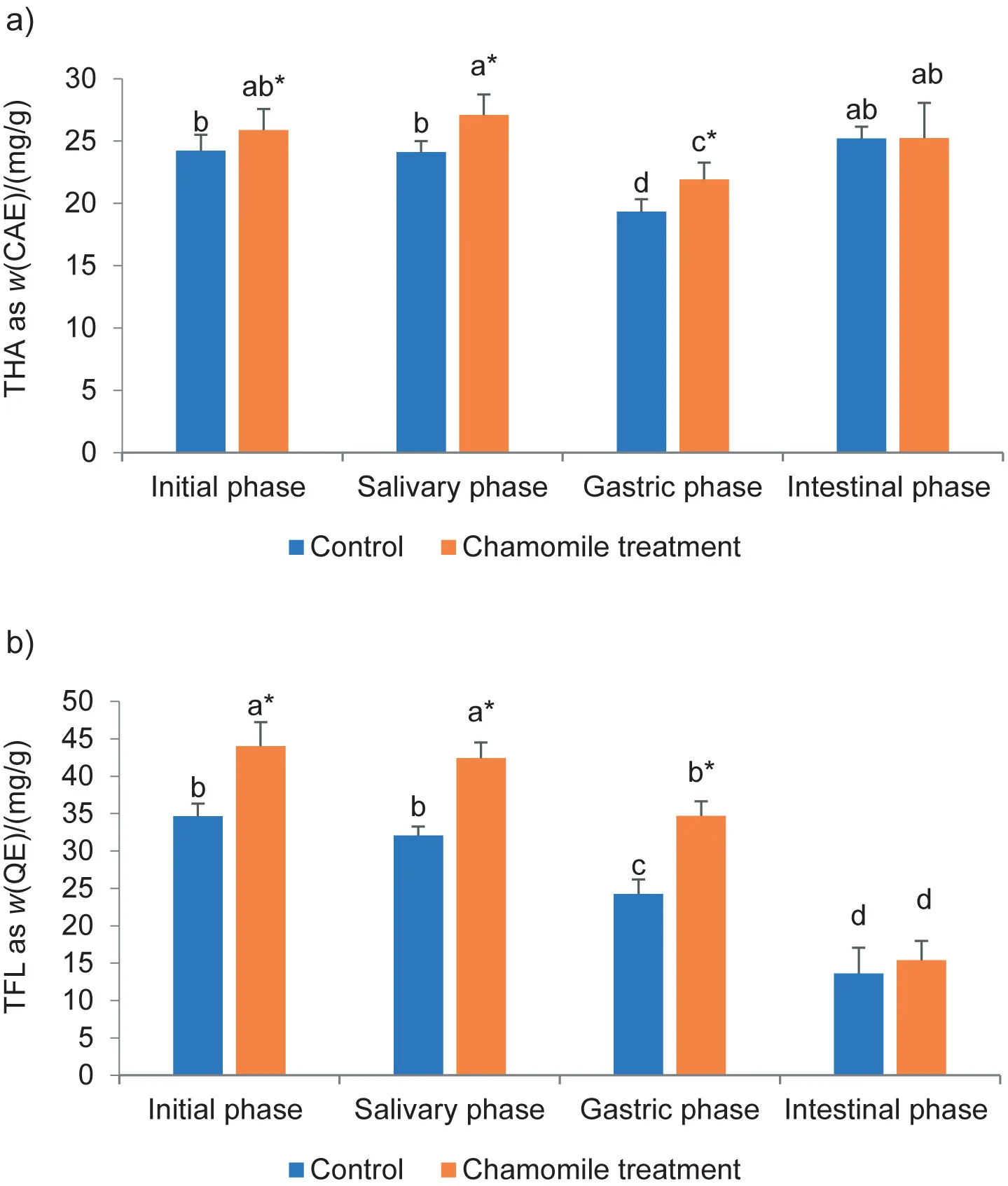
The content on dry mass basis of: a) total hydroxycinnamic acids (THA), and b) total flavonols (TFL) of control sample (deionized water) and treated sample (chamomile) of onion outer scales before (initial phase) and after (salivary, gastric and intestinal phase) *in vitro* digestion. Data are presented as mean value±S.D., *N*(onion bulb)=6. Different letters indicate significant differences at p≤0.05 for all samples and phases together. Asterisk indicates significant differences at p≤0.05 for each phase individually. CAE=caffeic acid equivalents, QE=quercetin equivalents

In the initial phase of digestion, the chamomile-treated sample (expressed as caffeic acid equivalents (CAE) on dry mass basis, 25.89 mg/g) had a statistically significantly higher THA value than the untreated control (24.23 mg/g) (Fig. 1a). During the oral phase, the highest THA value in the whole experiment was observed in the treated sample (27.08 mg/g), statistically significantly higher than in all other phases except for the treated sample in the initial phase and the control and treated samples in the intestinal phase. In the gastric phase, THA mass fractions generally decreased, especially in the control group (19.33 mg/g), where the lowest mass fraction of THA was measured, statistically significantly lower than in the other phases. In the treated sample in the gastric phase (21.93 mg/g), the mass fraction was slightly higher, but still lower than in the previous phases. In the intestinal phase, the THA mass fraction increased again in both the control (25.20 mg/g) and treated samples (25.24 mg/g), but without a statistically significant difference between them.

The TFL mass fraction (Fig. 1b), expressed as quercetin equivalents (QE) on dry mass basis, of the treated sample was significantly higher in the initial phase (44.01 mg/g) than in the control sample (34.63 mg/g). During the oral phase, there was a statistically insignificant decrease in flavonol mass fractions in the treated sample (42.40 mg/g), but the difference from the control (32.06 mg/g) remained statistically pronounced. In the gastric phase, a decrease in flavonoid mass fraction was observed in both groups, with values of 34.70 mg/g in the treated sample and 24.25 mg/g in the control sample. The lowest mass fractions of flavonols were measured in the intestinal phase in both samples: treated (15.38 mg/g) and control (13.60 mg/g), with no statistically significant difference between them.

The bioaccessibility of THA and TFL from onion outer scale extract in the control and chamomile-treated samples after the salivary, gastric and intestinal phases of *in vitro* digestion is shown in Fig. 2. High (>79.79 %) bioaccessibility of THA (Fig. 2a) was observed in the control and chamomile-treated samples in all phases of digestion. Statistically significantly higher THA bioaccessibility was found in the treated sample (105.52 %) than in the control sample (99.56 %) in the salivary phase of digestion. In the gastric phase, the treated sample (84.69 %) also had a statistically significantly higher percentage than the control onion scale sample (79.79 %). In this phase, there was a statistically significant decrease in bioaccessibility compared to the oral and intestinal phases.

**Fig. 2:**
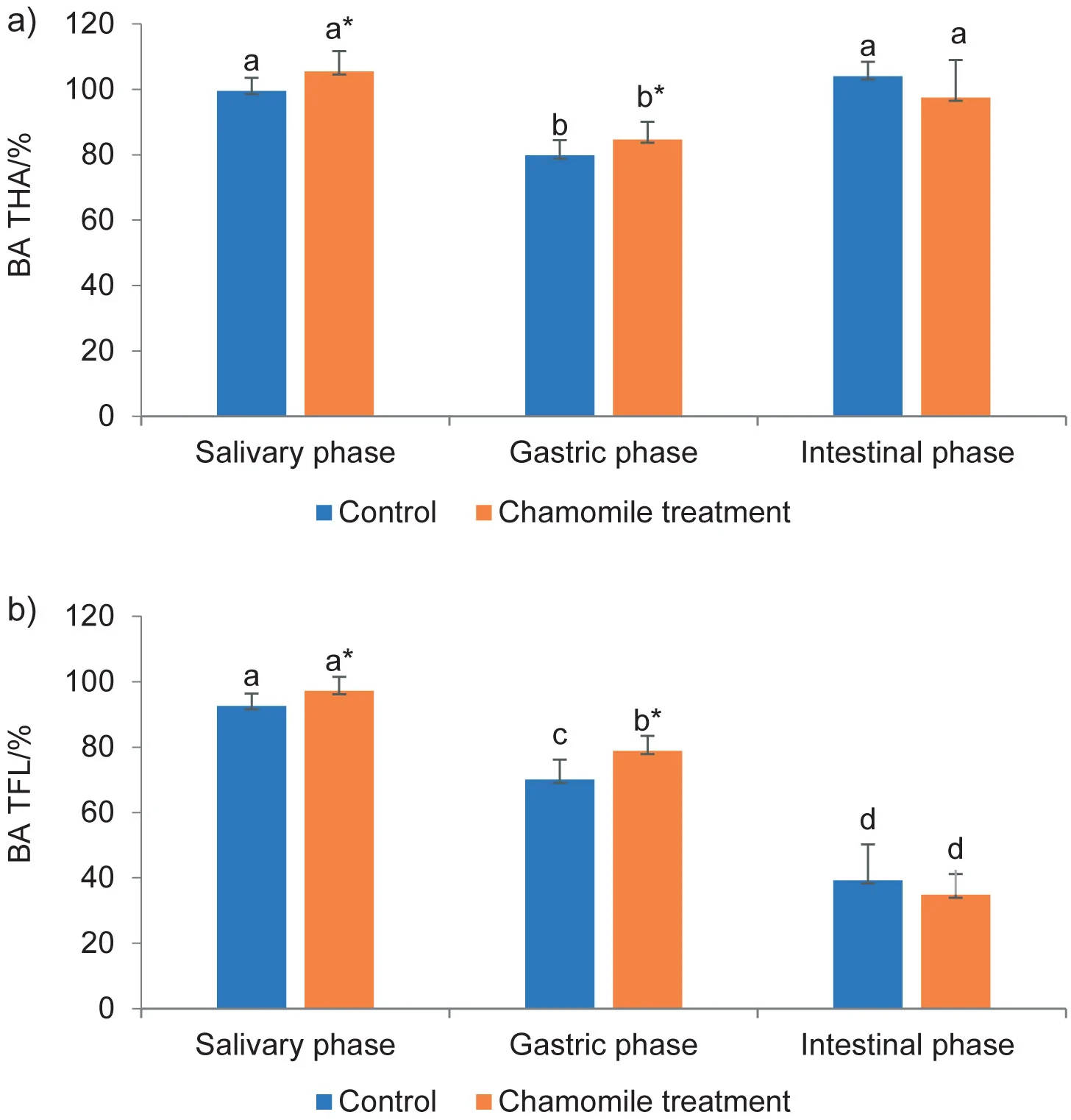
Bioaccessibility (BA) of: a) total hydroxycinnamic acids (THA), and of b) total flavonols (TFL) from the control sample (deionized water) and the treatment (chamomile) of onion outer scales after (salivary, gastric and intestinal phases) *in vitro* digestion. Data are presented as mean value±S.D, *N*(onion bulb)=6. Different letters indicate significant differences at p≤0.05 for all samples and phases together. Asterisk indicates significant differences at p≤0.05 for each phase individually

TFL bioaccessibility (Fig. 2b) of both samples (control and test) in the salivary and gastric phases of *in vitro* digestion was high (>70.01 %). In these two phases, the sample treated with chamomile (97.15 % in the salivary and 78.84 % in the gastric phase) had higher percentage of bioaccessibility than the control samples (92.58 % in the salivary and 70.01 % in the gastric phase). A statistically significant decrease was observed for both samples in the gastric phase compared to the oral phase. This decline continued in the intestinal phase, where bioaccessibility for both samples was significantly lower than in the first two phases of *in vitro* digestion.

Hydroxycinnamic acids are phenolic acid derivatives of cinnamic acid, and they include caffeic, ferulic, *p*-coumaric and sinapic acids (*3*). In plants, they help strengthen cell walls, defend against pathogens and oxidative stress, and support signalling, while in the human body they have antioxidant and hypoglycaemic effects, modulating enzymes involved in the breakdown of carbohydrates (*23*). In our experiment, in three of the four observed phases, a statistically significant increase in THA content can be observed, which indicates a positive impact of chamomile treatment. Chamomile is an excellent source of caffeic and ferulic acid derivatives (*24*) and, for this reason, chamomile treatment could act as a donor of these phenolic acids to the common onion, the acceptor plant. In the oral phase, the treated sample reached the highest mass fraction of THA in the entire experiment. This increase may indicate that hydroxycinnamic acids, often bound to polysaccharides and cell walls, are rapidly released by the action of amylase at neutral pH. Although short, this phase allowed the enzymes to release a significant amount of the bioactive molecules, as confirmed by the high bioaccessibility (105.52 %) in the oral phase of digestion. This was followed by the gastric phase, whose acidic environment often destabilises the more sensitive phenolic acids. In this case, this trend was evident: there was a decrease in the mass fraction of THA in both samples, but in the treated sample, as expected, statistically significantly higher values were maintained, which suggests the relative resistance of the transferred acids to gastric conditions. This resistance could result from interactions with onion plant matrices that protect them from degradation, which is also noted in similar papers on cinnamic acids in plant foods (*25*). In the intestinal phase, the THA stabilised. Although the values were slightly lower than in the oral phase, they remained at a mass fraction comparable to the initial one. According to Manach *et al*. (*26*), this may mean that the compounds that reached this stage were stable and potentially ready for absorption, or that there was partial degradation into smaller phenolic fragments that could have the same, or possibly even greater, biological activity. Overall, hydroxycinnamic acids showed relative stability and high bioaccessibility during digestion. In the treated sample, their profile indicates that interspecies transmission cannot only increase the initial content of these compounds, but also enable their more efficient release and greater stability, allowing them to "survive" digestive conditions. For the development of functional foods with a targeted effect on glucose metabolism, this information is of great importance.

Flavonoids are known for their strong antioxidant activity, the ability to bind free radicals, and their role in regulating key enzymes involved in glucose metabolism (*27*). In plants, they act as pigments, defence mechanisms and signalling molecules, and in the human body as silent allies in preserving homeostasis. It is therefore not surprising that flavonoids are the subject of numerous studies on functional nutrition. In this study, the analysis of total flavonols, as a subgroup of flavonoids, showed a significantly higher mass fraction in the treated onion than in the control sample, in all digestive phases except the intestinal phase, in which the increase was not statistically significant. During the oral phase, the mass fraction of TFL in the treated sample was still higher than in the control, which may indicate that chamomile flavonols are mostly in the free form and therefore quickly released, and more stable in a neutral pH environment, while endogenous onion flavonols can be bound to sugar or protein structures that still require degradation. In the gastric phase, where the pH is low (≈2), a statistically significant decrease in TFL in both samples is expected, but the values in the treated sample remained higher. This stage is known for its challenges to the stability of flavonoid compounds because many are sensitive to hydrolysis in an acidic medium, especially when present in the form of glycosides. However, the fact that they do not completely disappear supports the resistance of the transferred compounds, perhaps thanks to the protection provided by the onion plant matrix. Furthermore, the intestinal phase, a key stage for absorption, shows the lowest flavonol values throughout the model. The treated and control samples are statistically almost equal in mass fraction, and bioaccessibility is only 35 %. This result is not surprising, given the known instability of flavonoids at higher pH values (*28*). A higher pH in the intestine (≈7) does not, therefore, bring complete "relief" to flavonols. On the contrary, this phase represents a second wave of stress for these compounds, namely oxidation, conjugation or complete degradation, leading to a significant reduction in their absorption. A similar decreasing trend in bioaccessibility was observed for flavonols in ginkgo casein, ginkgo glucose, ginkgo olive oil and spinach lemon juice formulations (*17*, *21*). However, there are also experiments with opposite results. For example, the literature (*21*, *29*) reports an increase in flavonoid bioaccessibility in the intestinal phase in berry, green tea and ginkgo water extracts, which can be attributed to differences in the plant matrix and the structure of the flavonoids present (*21*). These discrepancies, however, confirm that the stability and utilisation of flavonols are highly contextual and depend on many factors, including the source of the compounds and the physicochemical properties of the digestive environment. The amount of flavonols through the digestive model is progressively reduced. In addition to the chemical delicacy of flavonoids, these data also indicate the importance of design in the development of functional products to protect flavonols during their journey through the digestive system, either through microencapsulation, the use of synergistic plant matrices or the selection of more resistant molecular forms. In the combination of onion and chamomile, the loss of distinctiveness between treatment and control in the intestinal phase suggests that the transfer of chamomile flavonols has been successful, but their stability in the later stages of digestion remains a challenge for future formulations.

ABTS and FRAP methods were used to evaluate the antioxidant activity of the control sample (deionized water) and the treated (chamomile) onion outer scales before (initial phase) and after (salivary, gastric and intestinal phases) *in vitro* digestion (Fig. 3). High antioxidant activity was observed with both methods. The percentage of ABTS radical inhibition ranged from 85.14 to 92.98 %, and the reduction percentage for the FRAP method ranged from 91.91 to 97.46 %. In the ABTS method, a statistically significantly higher value for the treated sample than for the control sample was observed only in the intestinal phase of *in vitro* digestion (91.51 *vs* 86.21 %). For the FRAP method, onions treated with chamomile showed statistically significantly higher values before (initial phase) and after (salivary, gastric and intestinal phases) *in vitro* digestion than the control onions treated with water.

**Fig. 3:**
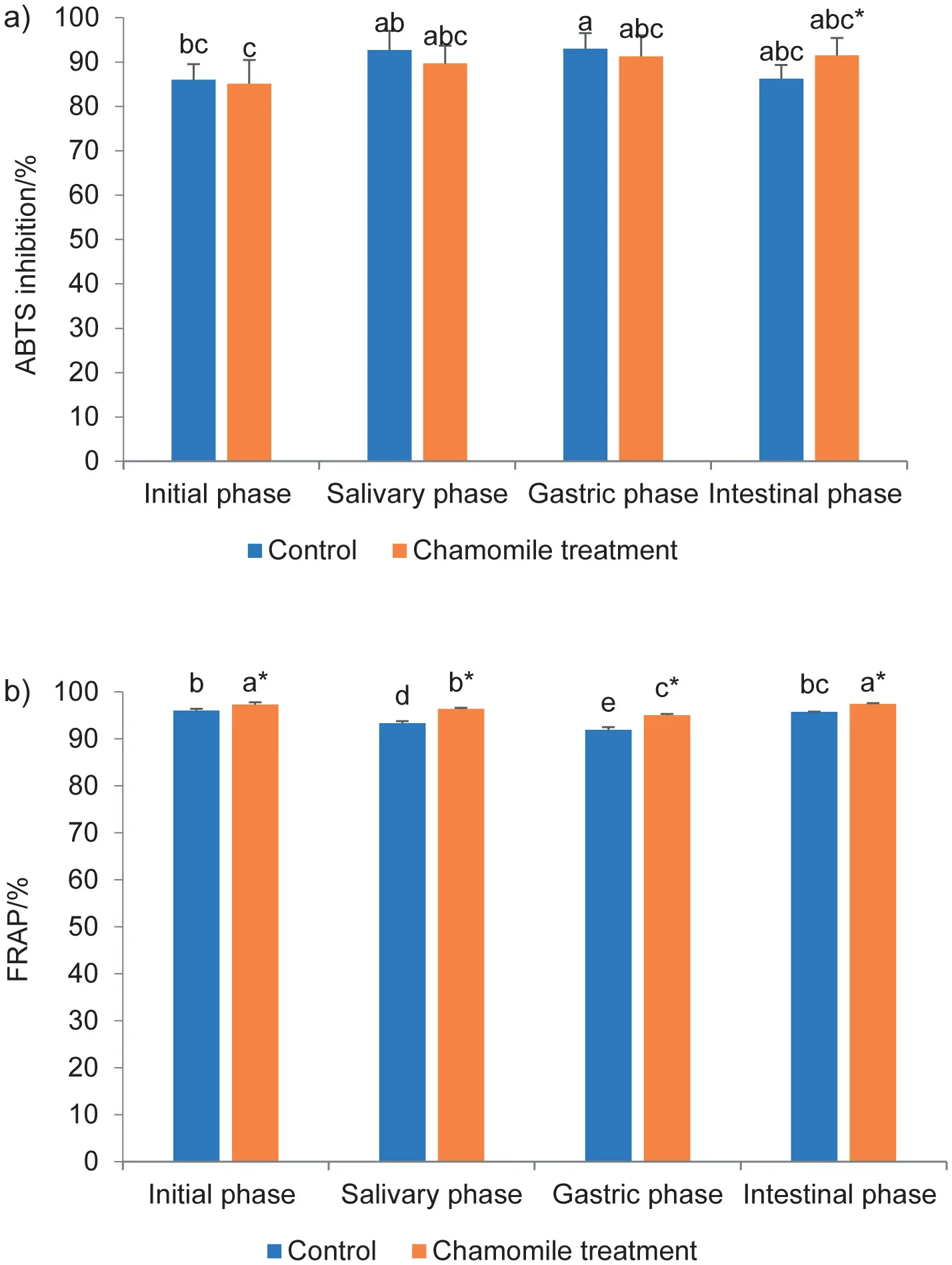
Antioxidant activity: a) ABTS and b) FRAP of control sample (deionized water) and treated (chamomile) onion outer scales before (initial phase) and after (salivary, gastric and intestinal phases) *in vitro* digestion. Data are presented as mean value±S.D, *N*(onion bulb)=6. Different letters indicate significant differences at p≤0.05 for all samples and phases together. Asterisk indicates significant differences at p≤0.05 for each phase individually. ABTS=2,2’-azinobis(3-ethylbenzothiazoline-6-sulfonic acid), FRAP=Fe(III) ion reducing antioxidant power

In our experiment, two antioxidant methods were used to evaluate the control sample (deionized water) and the treated (chamomile) onion outer scales before (initial phase) and after (salivary, gastric and intestinal phases) *in vitro* digestion. We combined the ABTS method, which can evaluate both hydrophilic and lipophilic antioxidants, with the FRAP method, which primarily measures hydrophilic antioxidants (*30*, *31*). Combining antioxidant activity methods reduces the limitations of each individual method. The DPPH method was not suitable for the measurement of our samples due to the purple colour of the onion outer scale extract, which affected the DPPH measurement. In both methods, onion outer scale samples before and after all stages of digestion showed high antioxidant activity (ABTS>85.14 % and FRAP>91.91 %). The high antioxidant activity of onion samples is in accordance with the literature data (*32*–*34*). In the FRAP method, a statistically significant increase was observed for all treated samples compared to control samples in all phases of *in vitro* digestion, and in the ABTS method only in the intestinal phase of *in vitro* digestion. This could be attributed to the high concentration of hydroxycinnamic acids and their high bioaccessibility in chamomile-treated onion samples. As mentioned previously, chamomile is an excellent source of hydroxycinnamic acids, especially caffeic and ferulic acids, with high antioxidant activity (*24*, *35*).

All treated samples (Fig. 4) had moderate antiglycation activity, ranging from 42.86 to 62.24 %. The control samples had weak (26.92 % initial phase) to moderate antiglycation activity (36.35–54.60 %). In the initial phase, the treated sample (43.52 %) showed a statistically significantly higher inhibition of bovine serum albumin (BSA) glycation than the control (26.92 %), indicating that the transfer of specialised metabolites from chamomile to onion had a positive effect on antiglycation properties. During the oral phase, activity increased further in control (36.35 %) and in the treated sample (49.85 %), with the difference in the percentage of inhibition being statistically significant. In the gastric phase, a decrease in inhibitory activity was observed in the treated sample, but it still maintained higher activity (42.86 %) than the control (36.98 %). In the intestinal phase, the highest percentage of inhibition of bovine albumin glycation was recorded in the treated sample (62.24 %), statistically significantly higher than in all other phases and samples, suggesting that bioactive compounds from treated onions not only remain stable under digestive conditions, but also potentially increase their bioaccessibility and efficacy at this stage.

**Fig. 4:**
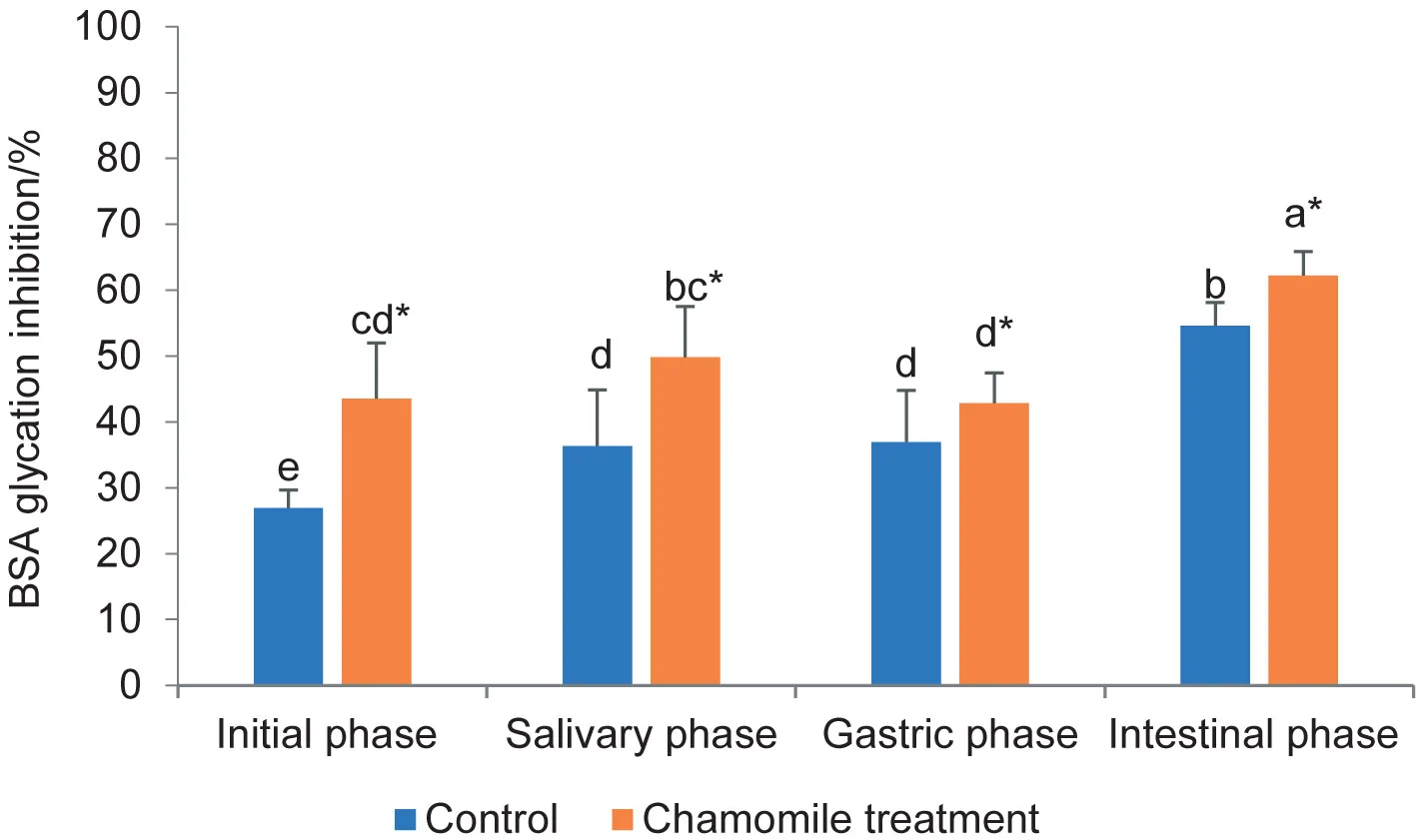
Antiglycation activity (BSA method) of control sample (deionized water) and treated (chamomile) onion outer scales before (initial phase) and after (salivary, gastric and intestinal phases) *in vitro* digestion. Data are presented as mean value±S.D, *N*(onion bulb)=6. Different letters indicate significant differences at p≤0.05 for all samples and phases together. Asterisk indicates significant differences at p≤0.05 for each phase individually. BSA=bovine serum albumin

In a metabolic disorder such as diabetes, there are not only elevated blood glucose levels but also a whole series of accompanying biochemical processes that damage cells and tissues over time. Among them, protein glycation occupies a special place. This process, also known as the Maillard reaction, is a non-enzymatic interaction between reducing sugars, such as glucose, and protein molecules (*36*). Sugars irreversibly bind to proteins, creating advanced glycation end products (AGEs), molecules that accelerate cell ageing, disrupt the elasticity of blood vessels, and activate inflammatory pathways (*37*), and cause nerve and retinal damage, cataracts, and chronic kidney disease (*36*). Because of these negative effects of glycation, it is crucial to identify compounds that can suppress this process and thus reduce the aforementioned diabetic complications. Consequently, the ability of plant extracts to inhibit glycation is increasingly used as an indicator of their antidiabetic potential.

Throughout this work, glycation suppression was investigated using the BSA method, assessing the ability of the test extract to prevent glucose binding to bovine serum albumin, which is used as an analogue of human albumin due to its structural and functional similarity. Overall, the results show that both control and treated samples of onion outer scales exhibited protective antiglycation activity. Treated samples had moderate antiglycation activity, while the control samples had weak to moderate activity. This activity was statistically significantly higher in the treated samples than in the control samples. In the initial phase, the control sample showed the lowest value among all samples. In the same phase, the inhibition of BSA in the treated sample was moderate, compared to the weak inhibition in the control sample. It is already evident that the compounds transferred from chamomile, known for its richness in flavonoids and phenolic acids, greatly contributed to the antiglycation effect. Given that apigenin and luteolin have already been confirmed as inhibitors of AGE formation, their presence may be a key factor in explaining the obtained results. During the oral phase, which is characterised by neutral pH and mild enzymatic activity, the inhibition of glycation further increases. It appears that most compounds are activated or released from the plant matrix, especially in the treated sample, where a statistically significant difference compared to the control sample is still maintained. The gastric phase showed the lowest inhibitory activity in the treated sample, which is expected, given the already mentioned sensitivity of certain phenolic compounds to low pH. Nevertheless, the treated sample retained a statistically significantly higher level of glycation inhibition than the control. From this, we can again conclude that, although there is a decline relative to the oral phase, the compounds transferred from chamomile extract to onions show a certain level of resistance, or are at least partially protected by the onion plant matrix, which mitigates their acid degradation.

The intestinal phase, as the final and most important stage in the context of absorption and the biological effect of compounds, yielded the highest percentage of BSA glycation inhibition, especially in the treated sample. This upward trend may indicate the cumulative effect of the release of compounds throughout the digestive process, as well as their stability and potential activation in the more alkaline environment of the small intestine. It is also possible that the compounds that reach this stage are more effective or reactive in the presence of BSA itself, and that new metabolites with antiglycation properties are formed during digestion. From the results of the experiment, it was already evident that the bioaccessibility of flavonols in the intestine was relatively low (35 %), so the high level of glycation inhibition at this stage suggests that these compounds are not the sole carriers of the antiglycation effect. Given that in the same digestive phase, the bioaccessibility of hydroxycinnamic acids was significantly higher (97 %), it could be the main contributor to this biological activity. Chlorogenic and ferulic acids have been shown to prevent the formation of AGE products through various mechanisms, including scavenging reactive carbonyl compounds and chelation of metal ions (*38*, *39*). It is possible, therefore, that a kind of "phenolic power transition" occurs in this part of digestion, where the dominant role is taken over by more stable and available compounds, whose synergy with residual flavonols further enhances the overall effect. The highest inhibition of BSA glycation at this stage therefore does not necessarily reflect the amount of one type of compound, but indicates the efficacy and cooperation of several groups of bioactive metabolites.

Fourier transform infrared (FTIR) spectroscopy is one of the most important non-destructive analytical techniques used to identify the functional groups of organic compounds and is widely used for quality control in the pharmaceutical industries. FTIR has also become increasingly useful for evaluating herbal quality (*40*–*43*).

The absorption spectra of dried onion outer scales and dried chamomile flower powder are shown in Fig. 5, FTIR spectra of pure phenolic compounds (apigenin, caffeic acid and quercetin) are presented in Fig. 6. Onion outer scales and chamomile flower powder are composed of polyphenolic compounds with O–H bonds (around 3400–3200 cm^-1^), C=O bonds (around 1670–1660 cm^−1^), and other characteristic peaks for C-C, C-H, C-O-C and C-O bonds around 1030-1023 cm^-1^ as recorded in the literature (*40*–*43*). The FTIR spectra of the onion outer scale control treatment had a broad peak at 3331 cm^−1^, the chamomile treatment at 3298 cm^−1^, and pure chamomile powder at 3293 cm^−1^. The region of 3331-3293 cm^-1^ indicates stretching of polymeric hydroxyl groups. O-H bonds are mainly derived from phenols and flavonoids (*44*).

**Fig. 5:**
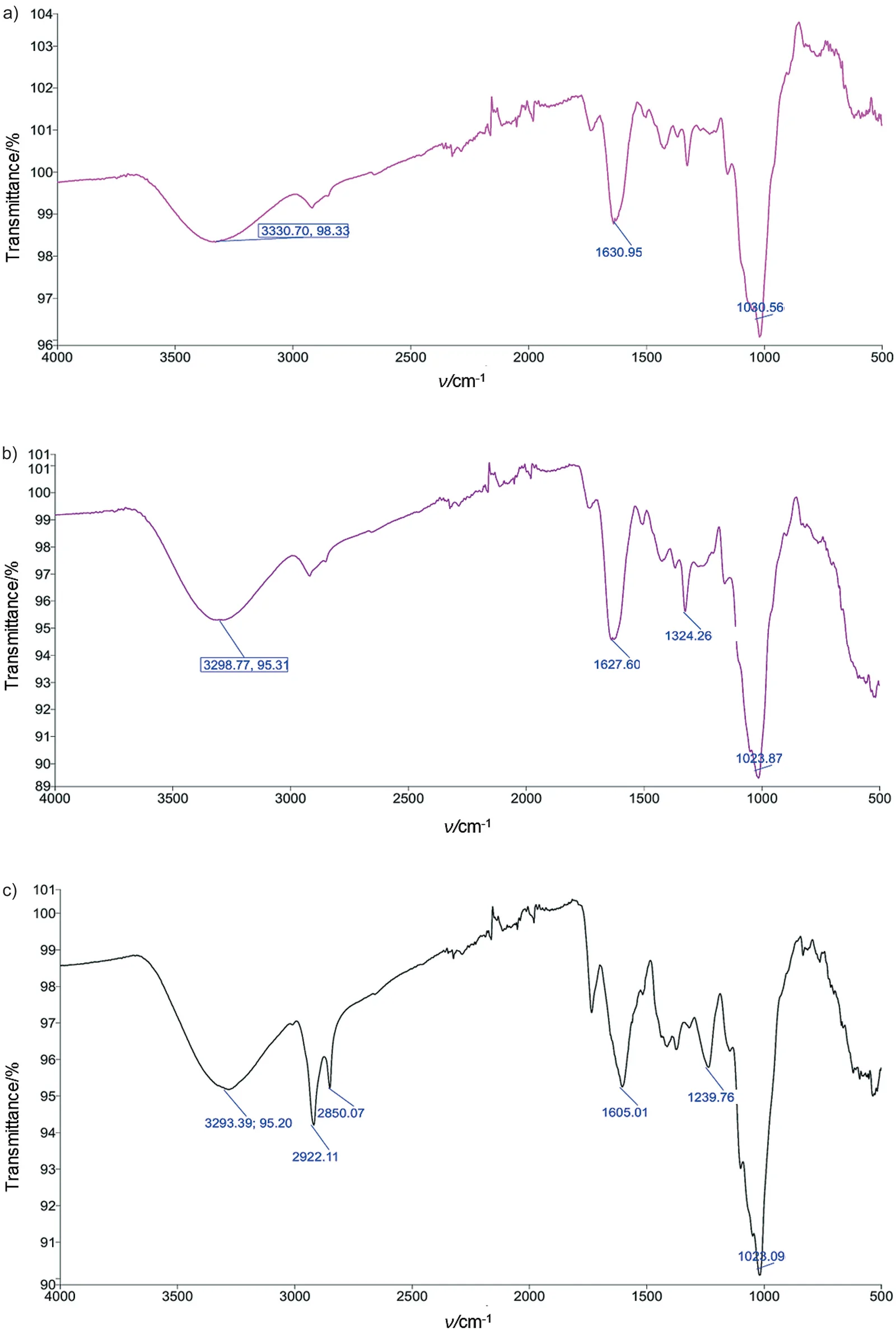
The FTIR spectra of: a) the onion outer scale control treatment, b) of the chamomile-treated onion outer scales and c) of the chamomile powder

**Fig. 6:**
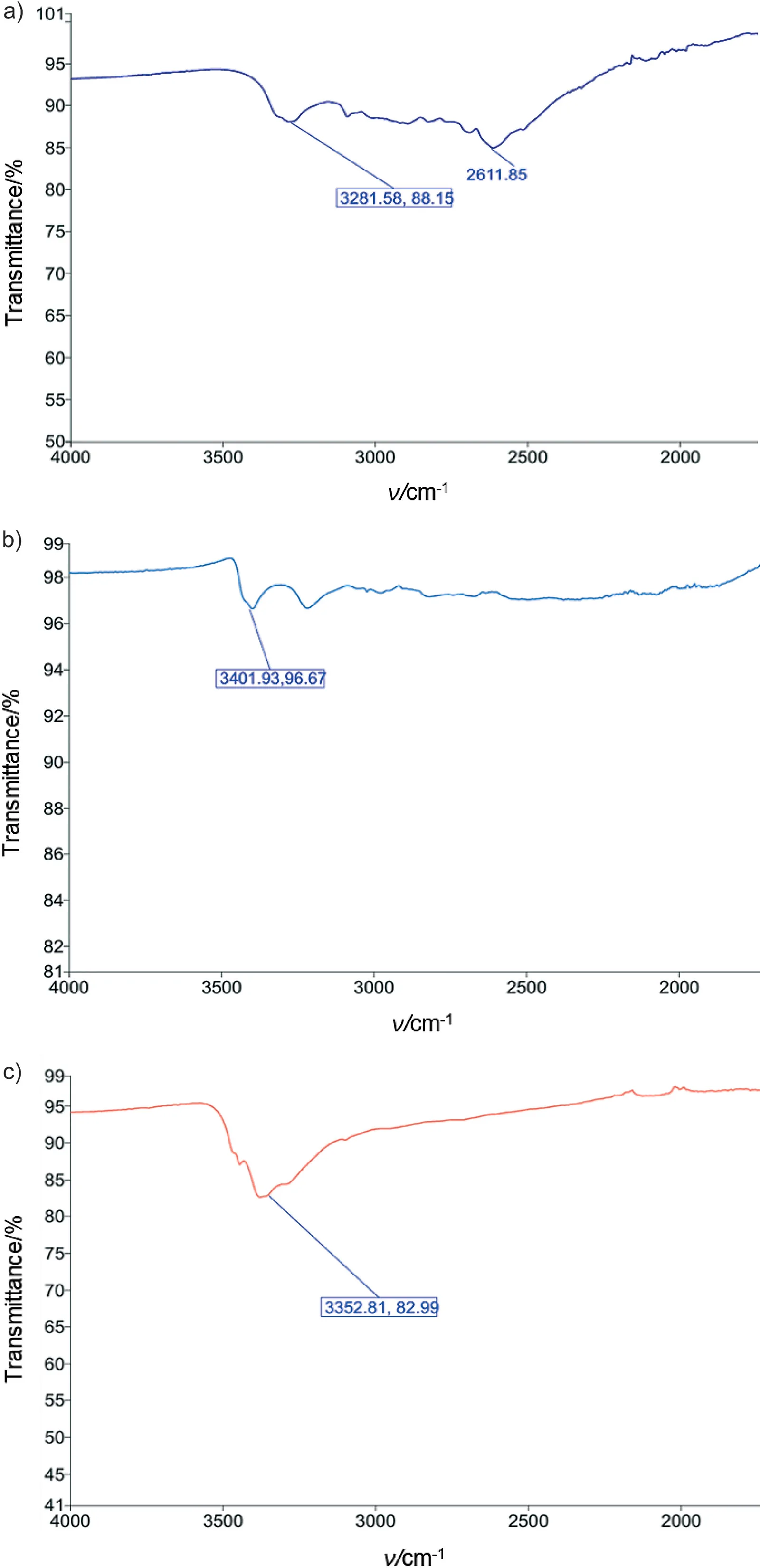
The FTIR spectra of: a) apigenin, b) caffeic acid and c) quercetin

Quercetin, the main flavonoid in onion, had a characteristic broad peak at 3353 cm^−1^ corresponding to its hydroxyl groups. The O-H groups of quercetin are located at C-3, C-3’, C-4’, C-5 and C-7, and this peak is in the range of 3600-3100 cm^−1^ (*45*). Apigenin, the main flavonoid in chamomile, has O-H groups at C-5, C-7 and C-4′ positions (*45*). This O-H group is located at 3282 cm^−1^ in the apigenin FTIR spectrum. As previously discussed, chamomile is an excellent source of caffeic acid (*24*). Caffeic acid has two O-H groups located at C-3 and C-4 positions. In the FTIR spectrum of caffeic acid, a visible peak is present at 3401 cm^−1^.

Transmittance of O–H bonds (around 3600-3100 cm^-1^) of the pure polyphenolic compounds was 82.99 % for quercetin, 88.15 % for apigenin, 96.67 % for caffeic acid, while for onion outer scales in the control treatment it was 98.33 %, for chamomile treatment 95.31 % and for pure chamomile powder 95.20 %. A higher number of O-H groups has a positive effect on antioxidant activity (*3*, *44*). The lowest transmittance was recorded for quercetin, the compound with five O-H groups, and the highest for caffeic acid, the compound with two O-H groups. The increase in THA, expressed as CAE on dry mass basis (control 39.20 mg/g, chamomile treatment 48.80 mg/g) and TFL, expressed as QE (control 51.75 mg/g, chamomile treatment 78.77 mg/g) measured by spectrophotometry (data not shown) was confirmed by FTIR analysis, which showed that the transmittance of the O–H group, mainly derived from polyphenols, was lower for the chamomile treatment than for the control onion scales samples. This indicates a positive effect of chamomile treatment on the polyphenolic composition of the outer onion scales and the antioxidant and antiglycation activity. Chamomile treatment could have facilitated the entry of apigenin and caffeic acid from chamomile tea, and may also have caused stress in the plant, resulting in additional synthesis of quercetin in the onion.

### Pearson’s correlations between polyphenolic content and bioaccessibility, antioxidant and antiglycation activity

Pearson’s correlations of total hydroxycinnamic acids (THA), total flavonols (TFL), bioaccessibility of total hydroxycinnamic acids (BA THA in %), bioaccessibility of total flavonols (BA TFL in %), antioxidant activity (ABTS and FRAP methods) and antiglycation activity (BSA method) in onion scale extracts (control and test treatment with chamomile extract) during simulated *in vitro* gastrointestinal digestion are presented in Table 1. Evans’ correlations coefficient from 0.00–0.19 indicates a very weak correlation, from 0.20–0.39 a weak correlation, from 0.40–0.59 a moderate correlation, from 0.60-0.79 a strong correlation and from 0.80–1.00 a very strong correlation (*46*). The highest positive correlations were observed between the THA and BA THA and between the TFL and BA TFL. These correlations are very strong, with a value of 0.95. A very strong positive correlation (0.91) was observed between the antioxidant (FRAP) and antiglycation (BSA) activity. Strong positive correlations were observed between the FRAP and THA (0.79), BSA and THA (0.64), and FRAP and BA THA (0.62), while a moderate positive correlation was observed between BA THA and BSA (0.52). According to our results of *in vitro* digestion of onion outer scale extract, hydroxycinnamic acids significantly contribute to the antioxidant and antiglycation activity, which is in line with the literature (*10*, *17*, *19*–*21*, *47*–*49*).

**Table 1:** Correlations of total hydroxycinnamic acids (THA), total flavonols (TFL), bioaccessibility of the total hydroxycinnamic acids (BA THA), total flavonols (BA TFL), antioxidant activity (ABTS and FRAP methods) and antiglycation activity (BSA method) in onion extracts (control and chamomile extract treatment) during simulated *in vitro* gastrointestinal digestion

Variable	*w*(THA)/(mg/g)	*w*(TFL)/(mg/g)	BA THA/%	BA TFL/%	ABTS/%	FRAP/%	BSA/%
*w*(THA)/(mg/g)	1.00						
*w*(TFL)/(mg/g)	0.09	1.00					
BA THA/%	0.95	0.00	1.00				
BA TFL/%	-0.04	0.95	-0.05	1.00			
ABTS/%	-0.55	0.31	-0.62	0.40	1.00		
FRAP/%	0.79	-0.15	0.62	-0.41	-0.51	1.00	
BSA/%	0.64	-0.50	0.52	-0.71	-0.55	0.91	1.00

### PCA of the polyphenol group and antioxidant and antiglycation activity

Visualisation of the similarity and diversity of samples and parameters based on distances in the diagram is possible with principal component analysis (PCA) (*17*, *21*, *47*, *49*). The PCA diagram shows the measured polyphenols (THA and TFL), the bioaccessibility of THA and TFL, antioxidant activity (ABTS and FRAP) and antiglycaemic activity (BSA) of onion scale extracts (control and test treatment with chamomile during simulated *in vitro* gastrointestinal digestion).

The first (Factor 1) and second (Factor 2) principal components accounted for 57.20 and 28.78 %, respectively (Fig. 7). Together, the first two factors represented 85.98 % of the total variability. In the upper left quadrant are the test treatment samples from the salivary phase of *in vitro* digestion and the THA, BA THA and FRAP. On the same side, but in the lower quadrant, both samples (C=control and T=test treatment with chamomile) from the intestinal phase are located with BSA. Control samples from the salivary phase of digestion are located in the upper right quadrant, and control samples from the gastric phase of digestion are located in the lower right quadrant. Treatment samples from the gastric phase of digestion are located on the border between the upper and lower quadrants on the right side. In the test treatment in the salivary phase of *in vitro* digestion, the highest values of THA, BA THA, FRAP, TFL and BA TFL were measured. The test samples from the intestinal phase of *in vitro* digestion had the highest values of BSA and FRAP, which is clearly visible when looking at the distances between the samples and the measured parameters on the PCA diagram.

**Fig. 7:**
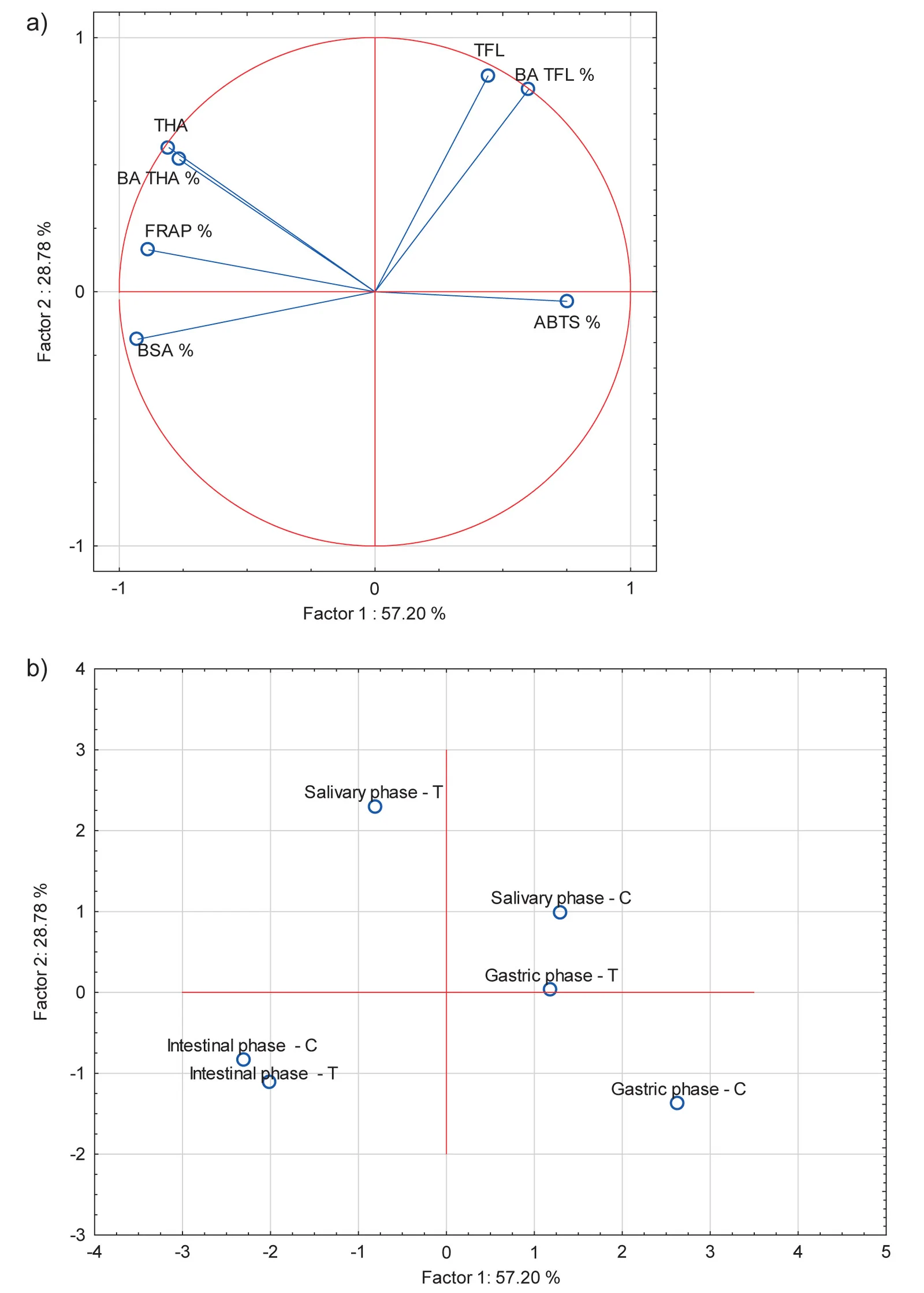
Principal component analysis (PCA) diagram of the measured polyphenols (total hydroxycinnamic acids (THA), total flavonols (TFL), bioaccessibility of the total hydroxycinnamic acids (BA THA) and of the total flavonols (BA TFL)), antioxidant activity (ABTS and FRAP) and antiglycaemic activity (BSA method) of onion scale extracts (C=control and T=test treatment with chamomile during simulated *in vitro* gastrointestinal digestion): a) grouping of analysed parameters, b) grouping of samples

## CONCLUSIONS

The hypothesis that the polyphenolic content and biological (antioxidant and antiglycation) activity of onion scales can be improved by simple immersion of onion root in chamomile extract was confirmed. The analysis showed an increase in the content of flavonols and hydroxycinnamic acids (initial, oral and gastric digestion phase), as well as the preservation of their bioaccessibility during simulated digestion, with the exception of flavonols in the intestinal phase. Antioxidant activity was also improved in the FRAP method before (initial phase) and after (salivary, gastric and intestinal phases) *in vitro* digestion for onions treated with chamomile and in the ABTS method only in the intestinal phase of *in vitro* digestion. In addition, extracts of chamomile-treated onions showed higher inhibitory activity on BSA glycation, indicating a potentially stronger antidiabetic effect. Treatment of onions with chamomile water extract is a good method of enrichment of biological waste, as confirmed by FTIR analysis. Onion scales represent a good source of bioaccessible polyphenols with high antioxidant and moderate antiglycation activity.

The enrichment of the phytochemical composition and biological potential of onions builds on recent research into the horizontal transfer of natural products and raises new questions about the potential applications of this technique for health and nutritional purposes. Since the method does not require genetic modification but relies on natural absorption mechanisms and stress induction, it could become a valuable tool for enriching the nutritional and therapeutic composition of herbs. These results provide a foundation for future research, which should further elucidate the mechanisms of transmission, long-term effects, and potential industrial applications of this phenomenon.
